# Mesenchymal WNT-5A/5B Signaling Represses Lung Alveolar Epithelial Progenitors

**DOI:** 10.3390/cells8101147

**Published:** 2019-09-25

**Authors:** Xinhui Wu, Eline M. van Dijk, John-Poul Ng-Blichfeldt, I. Sophie T. Bos, Chiara Ciminieri, Melanie Königshoff, Loes E.M. Kistemaker, Reinoud Gosens

**Affiliations:** 1 Department of Molecular Pharmacology, Faculty of Science and Engineering, University of Groningen, Antonius Deusinglaan 1, 9713AV Groningen, The Netherlands; x.wu@rug.nl (X.W.); aanelien@gmail.com (E.M.v.D.); jpblich@mrc-lmb.cam.ac.uk (J.-P.N.-B.); I.S.T.Bos@rug.nl (I.S.T.B.); c.ciminieri@rug.nl (C.C.);; 2 Groningen Research Institute for Asthma and COPD, University Medical Center Groningen, University of Groningen, 9700 RB Groningen, The Netherlands; 3 Division of Pulmonary Sciences and Critical Care Medicine, School of Medicine, University of Colorado, Aurora, CO 80045, USA; melanie.koenigshoff@ucdenver.edu; 4 Aquilo BV, 9713 AV Groningen, The Netherlands

**Keywords:** chronic obstructive pulmonary disease (COPD), alveolar repair, WNT-5A, WNT-5B, WNT signaling pathway, precision-cut-lung slices (PCLS), lung organoids

## Abstract

Chronic obstructive pulmonary disease (COPD) represents a worldwide concern with high morbidity and mortality, and is believed to be associated with accelerated ageing of the lung. Alveolar abnormalities leading to emphysema are a key characteristic of COPD. Pulmonary alveolar epithelial type 2 cells (AT2) produce surfactant and function as progenitors for type 1 cells. Increasing evidence shows elevated WNT-5A/B expression in ageing and in COPD that may contribute to the disease process. However, supportive roles for WNT-5A/B in lung regeneration were also reported in different studies. Thus, we explored the role of WNT-5A/B on alveolar epithelial progenitors (AEPs) in more detail. We established a Precision-Cut-Lung Slices (PCLS) model and a lung organoid model by co-culturing epithelial cells (EpCAM^+^/CD45^-^/CD31^-^) with fibroblasts in matrigel in vitro to study the impact of WNT-5A and WNT-5B. Our results show that WNT-5A and WNT-5B repress the growth of epithelial progenitors with WNT-5B preferentially restraining the growth and differentiation of alveolar epithelial progenitors. We provide evidence that both WNT-5A and WNT-5B negatively regulate the canonical WNT signaling pathway in alveolar epithelium. Taken together, these findings reveal the functional impact of WNT-5A/5B signaling on alveolar epithelial progenitors in the lung, which may contribute to defective alveolar repair in COPD.

## 1. Introduction

Chronic obstructive pulmonary disease (COPD) represents a worldwide concern with high morbidity and mortality, and is believed to be driven by tobacco smoke and other environmental or occupational exposures. COPD is a disease of the ageing lung, and the interactions between environmental exposures and accelerated ageing in COPD have been proposed [1,2,3]. The key problem underlying COPD pathogenesis is defective tissue repair in the airway and alveolar compartment, causing bronchitis and small airway remodeling on one hand and emphysema on the other. The lung interfaces with the environment across a continuous epithelium that is lined by various cell types along the proximal and distal airways [4,5,6]. The turnover rate of lung epithelia cells during homeostasis is normally low, however, in response to injuries, different lung stem/progenitor cells proliferate and differentiate rapidly to repair the damaged structures in order to maintain lung functions at different locations along the respiratory tree [7,8]. In the distal lung, club cells (expressing secretoglobin family 1A member 1, Scgb1a1) residing in bronchioles are progenitors for the repair of bronchiolar epithelium; AT2 cells, which express pro-surfactant protein C (pro-SPC), are the progenitors of AT1 cells that cover more than 90% of the alveolar area [8,9,10]. It has been illustrated recently that club and AT2 cells are replenished by BASCs (Bronchioalveolar stem cells), a joint stem/progenitor population, which are located at bronchioalveolar duct junctions (BADJs) [11,12]. Thus, the dynamic of the stem cell population is key to epithelial homeostasis.

Emerging studies show that impaired epithelial homeostasis is a main contributor in COPD pathophysiology [13,14,15]. Currently, most pharmacologic medications such as bronchodilators are only able to control symptoms or reduce the frequency and severity of exacerbations. However, none of the existing therapies for COPD have been shown to modify the long-term decline of lung function. Thus, regenerative pharmacological approaches targeting the pulmonary epithelium may be a promising strategy for lung regeneration in COPD.

Molecular signaling pathways that are essential regulators for alveolar progenitors to trigger respiratory epithelium repair include several secreted factors, such as Fibroblast Growth Factor (FGF)-10 signaling, the Notch pathway, Transforming Growth factor (TGF)-β signaling and WNT signaling [11,16,17,18,19,20,21,22]. These secreted factors and their pathways may yield potential drug targets. Accumulating evidence suggests that the WNT signaling pathway plays central roles in stem cell self-renewal, proliferation and regeneration during embryogenesis, tissue homeostasis, and wound repair in many organs including the respiratory system [23,24,25,26]. 19 Different WNT ligands exist in the human genome with distinct expression and signaling characteristics. These WNT ligands variably induce the activation of the main canonical β-catenin dependent pathway that regulates stemness and cell proliferation or non-canonical β-catenin independent pathways such as those linked to actin cytoskeletal organization. WNT-3A is known as a typical activator of canonical WNT/β-catenin signaling whereas WNT-5A is typically associated to non-canonical WNT/β-catenin independent signaling [27,28,29]. The aberrant activity of the WNT signaling pathway, especially the imbalance between canonical and non-canonical WNT signaling, has been widely demonstrated in different diseases associated with ageing, including COPD. Baarsma et al. showed that WNT-5A derived from fibroblasts was increased in expression in both experimental COPD models and in human COPD specimens [30]. WNT-5A negatively affects canonical WNT signal-driven alveolar epithelial cell function, thereby contributing to disease development and progression. Likewise, increased WNT-5B expression in COPD has been linked to pro-inflammatory signaling functions [31,32] However, A. Nabhan et al. recently demonstrated that WNTs including WNT-5A expressed by the fibroblasts activate the canonical WNT signaling pathway in neighboring AT2 cells to facilitate regeneration [33].

WNT-5A and WNT-5B are two structurally closely related WNT ligands; however, the roles of WNT-5A and WNT-5B signaling in alveolar repair are debated and incompletely identified [27,29,30,31,34,35]. In this study, we aimed to investigate the role of WNT-5A and WNT-5B further to identify the specific roles of these two non-canonical WNT ligands on epithelial progenitors. Our results show that WNT-5A and WNT-5B repress the growth of epithelial progenitors with WNT-5B preferentially restraining the growth and differentiation of alveolar epithelial progenitors. Ex vivo co-culture organoid of murine fibroblasts and EpCAM^+^ cells from TCF/Lef:H2B-GFP mice treated with WNT-5A/5B revealed that both WNT-5A and WNT-5B negatively regulate the canonical WNT signaling pathway in alveolar epithelium, suggesting that targeting the canonical to non-canonical WNT signaling imbalance could be a new approach to prevent defective lung repair.

## 2. Materials and Methods

### 2.1. Antibodies and Reagents

Recombinant Human/Mouse WNT-5A and Recombinant Human/Mouse WNT-5B were obtained from R&D Systems Inc. (Minneapolis, MN, USA). Rabbit anti-Prosurfactant Protein C (proSP-C) was purchased from EMD Millipore Corporation (Amsterdam-Zuidoost, North Holland, The Netherlands). Mouse anti-acetylated α tubulin was purchased from Santa Cruz Biotechnology Inc. (Heidelberg, Germany). Mouse anti E-cadherin was purchased from BD Biosciences (Bedford, MA, USA). Matrigel and cell culture inserts were obtained from Corning Incorporated (New York, NY, USA). CD31, CD45, and CD326 (EpCAM) microbeads were purchased from Miltenyi Biotec (Leiden, The Netherlands).

### 2.2. Animals

C57BL/6J (555) mice and TCF/Lef:H2B-GFP mice (both genders, aged from 8–12 weeks) were used in this study. Animals were housed under a 12 h light/dark cycle with controlled humidity and room temperature at 24 ± 1 °C. Water and food were provided ad libitum. All experiments were performed according to the national guidelines and upon approval of the experimental procedures by the local Animal Care and Use committee of the University of Groningen.

### 2.3. Precision-cut Lung Slices

Precision-cut lung slices were prepared as described previously [36]. The mice were euthanized by subcutaneous injection of ketamine. A small incision was made on the trachea to insert the cannula. Subsequently, the lung was inflated through the cannula with a low melting-point agarose solution. Ice was used to cover the mouse for 20 min to allow the agarose to solidify within the lung, and then the lung was harvested. The lung was separated into different lobes before putting into the slicing machine. A tissue slicer (Leica VT 1000 S Vibrating blade microtome, Leica Biosystems B.V., Amsterdam, The Netherlands) was used to cut lung slices. The lung slices were all the same thickness (250 µm) for further experimental procedures. The lung slices were then incubated in a humidified atmosphere under 5% CO_2_/95% air at 37 °C. Every 30 min, slices were washed (four times in total) using the incubation medium. Lung slices were placed in the incubation medium and cultured at 37 °C in a humidified atmosphere under 5% CO_2_/95% air in 12-well culture plates, using three to four slices per well. The lung slices are viable for at least 3 days, as it has previously been demonstrated by our group that mitochondrial activity did not change.

### 2.4. mRNA Isolation and Real-Time PCR

Total RNA was extracted from PCLS with the Maxwell 16 LEV simply RNA tissue kit (Promega, Madison, WI, USA) according to the manufacturer’s instructions. The Reverse Transcription System (Promega, Madison, WI, USA) was used to reverse transcribe total RNA (1 µg) into cDNA. One µL diluted cDNA (1:20) was subjected to the Illumina Eco Personal QPCR System (Westburg, Leusden, The Netherlands) using FastStart Universal SYBR Green Master (Rox) from Roche Applied Science (Mannheim, Germany). The cycle parameters used in real-time PCR system were denaturation at 95 °C for 30 s, annealing at 59 °C for 30 s and extension at 72 °C for 30 s for 40 cycles, followed by 5 min at 72 °C. The amount of target genes was normalized to the housekeeping genes 18S Ribosomal 5 (18S), β-2 microglobulin (B2M), and ribosomal protein L13A (RPL13). Total RNA extracted from fibroblasts fractions or EpCAM^+^ cell fractions were performed by the Trizol method. LinRegPCR analysis software was used to analyze data. Primer sets used to analyze gene expression are shown in Table S1.

### 2.5. Western Blot

Slices preserved at –80 °C were thawed on ice, cold lysis buffer was added to each sample and samples were homogenized by ultrasonication. All samples were centrifuged at 12,000 rpm, 20 min, 4 °C, and the supernatant was collected. Protein concentrations were determined using the Pierce BCA Protein Assay Kit (Thermo Fisher Scientific, Landsmeer, The Netherlands) and subsequently subjected to SDS-PAGE, using 5% and 12% running gels (depending on protein size). Separated proteins were transferred to nitrocellulose membranes (0.45 µm), which were then blocked with ROTI solution for 1 h at room temperature. After blocking, the membrane was incubated with primary antibodies (anti-proSPC, 1:200; anti-β-actin, 1:500; anti-acetylated-α-tubulin, 1:200) at 4 °C overnight. Before and after the incubation of the second antibodies, the membrane was washed with 1 X TBST buffer for 10 min, three times. Finally, the membrane was developed using ECL reagents in the G-box system by SynGenesnap software (GeneSys 1.6.9, Cambridge, UK). Image J software (Java 8, open resource, NIH, US) was used to analyze the band intensity. Antibodies used are shown in Table S2.

### 2.6. Cell Culture

CCL206 mouse fibroblasts (Mlg [CCL206], ATCC) were cultured in DEME/F12 medium supplemented with 10% (*v*/*v*) fetal bovine serum (FBS), 100 U/mL penicillin/streptomycin, 2 mM L-glutamine, and 1% amphotericin B within a humid atmosphere under 5% CO_2_/95% air at 37 °C. MCR5 human fibroblasts were cultured in Ham’s F12 medium with the same supplementary ingredients as CCL206. Human alveolar epithelial cells (A549) were cultured in DMEM/F12 medium supplemented with 10% (*v*/*v*) FBS and antibiotics.

For organoid experiments, before the fibroblasts were mixed with EpCAM^+^ cells, CCL206/MCR5 were proliferation-inactivated by incubation in mitomycin C (10 μg/mL, Sigma, M4287) for 2 h, followed by 3 times PBS washing and then the cells were trypsinized before using to the organoid co-cultures. For the pre-treatment study, CCL206 were grown to 80% confluence in 6-well cell culture plates and were starved by normal culture medium but with 0.5% FBS supplemented; after 24 h starvation, the cells were incubated with either vehicle, recombinant WNT-5A, or recombinant WNT-5B within the normal culture medium for 24 h, and afterwards, they could be used for organoid culture.

### 2.7. Organoid Assay

The organoid assay is based on previously published protocols from our group [37]. In brief, the method is a co-culture of mouse/human lung epithelial cells (CD31^−^/CD45^−^/EpCAM^+^) and mouse/human lung fibroblasts (Mlg [CCL206], ATCC/MRC5) in Matrigel^®^ (Corning Life Sciences B.V., Amsterdam, The Netherlands). EpCAM^+^ cells were isolated from mouse (young adult mice) lung tissue without the trachea or human lung biopsies using the QuadroMACS™ Separator (Miltenyi Biotec, Leiden, The Netherlands). EpCAM^+^ cells (20,000) and 20,000 fibroblasts (CCL206 or MCR5) were mixed and suspended in 100 µL of Matrigel (BD Biosciences) prediluted 1:1 (*v*/*v*) with DMEM supplemented with 10% FBS were added to a 24-well Falcon^®^ cell culture insert (Corning, USA) in a 24-well plate containing 400 µL of organoid media. (DMEM/F-12 with 5% FBS, 1% penicillin/streptomycin, 1% glutamine, 1% amphotericin B, 0.025‰ EGF, 1% insulin-transferrin-selenium, 0.01% cholera toxin, and 1.75 ‰ bovine pituitary extract) underneath the insert. Cultures were incubated in a humid atmosphere under 5% CO_2_/95% air at 37 °C and medium in the well was refreshed every 2–3 days. To quantify the number of the organoids, light microscopy with 20× magnification is used manually and the diameter of the organoids (organoid size) were measured via NIS-Elements software with a light microscope.

### 2.8. Immunofluorescence

Organoids were fixed in acetone diluted 1:1 (*v*/*v*) with methanol for 15 min at –20 °C. After fixation, one mL of PBS with 0.02 % sodium azide was added to the well beneath the insert and 150 µL 5% BSA was added on top of the insert. The organoids were kept at 4 °C for one week after fixation. The BSA media was removed and the primary antibody incubation was performed in PBS buffer with 0.1% BSA and 0.1% Triton X-100 overnight at 4 °C. The next day, the organoids were washed with PBS for 30 min, three times and the secondary antibody incubation was performed overnight again at 4 °C. On the third day, the organoids were washed with PBS for 15 min. The organoids on the insert membrane were transferred to a glass slide with two drops of the mounting medium containing DAPI (Abcam 104139, Cambridge, UK), and then the coverslip was applied. The slides were kept at 4 °C. Confocal images were acquired using a Leica SP8 microscope at 63× magnification.

For the lung slices staining experiment, the frozen murine lung tissues were sectioned into the thickness of 5 μm and after 30 min drying process, the sections were fixed in acetone diluted 1:1 (*v*/*v*) with methanol for 10 min at –20 °C. Next, all sections were rinsed 3 times by cyto-TBS (CTBS), and then they were blocked within CTBS with 1% BSA and 2% normal donkey serum for 30 min. The primary antibodies incubation (anti-vimentin, 1:100 dilution, dako M0725; WNT5A antibody, 1:50 dilution, R&D system AF645; WNT5B antibody, 1:50 dilution, Santa Cruz Sc109464) were performed in the CTBS buffer contains 1% BSA and 2% normal donkey serum overnight at 4 °C. The next day, the lung sections were washed by CTBS for three times and the secondary antibody incubation (DAM 488, 1:2000 dilution, Life tech. corp. A21202; DAG 568, 1:2000 dilution, Life tech. corp. A11057) was performed for 2 h at RT. Washed 3 times with CTBS, the sections were transferred to a glass slide with two drops of the mounting medium containing DAPI, and then the coverslip was applied. Leica 4000b microscope was used to visualize the immunofluorescence. Images were obtained with LASX (Leica) software (open resource, Leica Microsystems GmbH, Wetzlar, Germany).

### 2.9. Wnt/β-catenin Activity Assay

The TOP/FOP flash assay was performed based on the protocol [30]. A549 cells were seeded in 96-well plate. When confluent, cells were transfected with 100 ng/well of either TOP luciferase reporter plasmid or the negative control FOP plasmid using Lipofectamine™ LTX Reagent with PLUS™ Reagent (Invitrogen, Carlsbad, US) in serum-free Opti-MEM^®^ medium (Life Technologies, Carlsbad, US). After 5 h transfection, cells were stimulated with either vehicle, WNT-5A (50 ng/mL), or WNT-5B (50 ng/mL) in Opti-MEM^®^ medium supplemented with 0.1% FBS. After stimulation, cells were lysed by the Bright-Glo™ Luciferase Assay System (Promega). Subsequently, the luciferase activity was measured using a Synergy HTX Multi-Mode Microplate Reader (BioTek, Winooski, US). Data were collected with Gen5 software (BioTek).

### 2.10. Statistical Analysis

All data are presented as mean ± SEM within the text. The paired *t*-test or one-way ANOVA test were used to assess the statistical significance, unless otherwise stated. The *p*-values indicating statistically significant differences between the mean values are defined as follows: * *p* < 0.05, ** *p* < 0.01, and *** *p* < 0.001. All statistical procedures were performed with GraphPad Prism 5 software.

## 3. Results

### 3.1. WNT-5A and WNT-5B Are Enhanced in Ageing Mice

**Figure 1:**
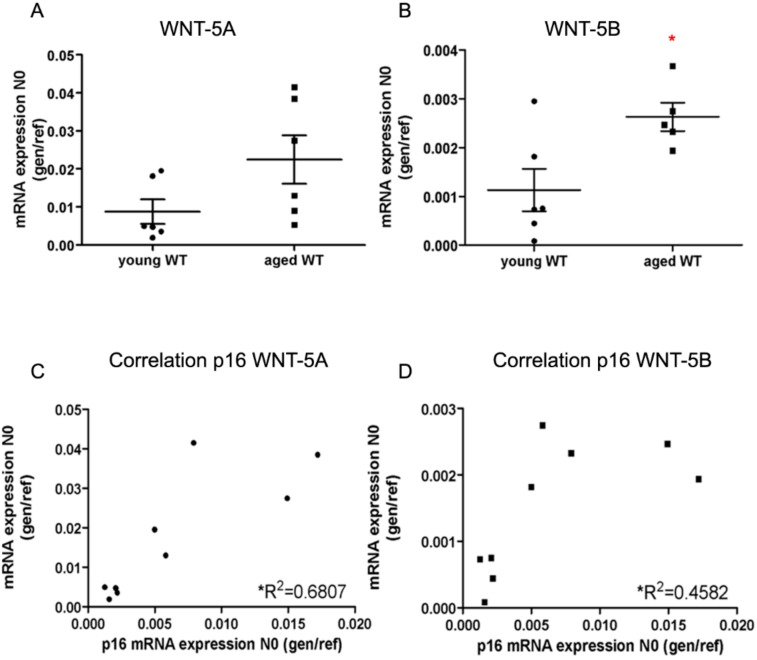
WNT-5A and WNT-5B increased in old mice. (**A**) The gene expression level of WNT-5A in young wild type mice (left) and aged mice (right). (**B**) The gene expression level of WNT-5B in young wild type mice (left) and aged mice (right). (**C**) Significant correlation between WNT-5A and p16 mRNA expression. (**D**) Significant correlation between WNT-5B and p16 mRNA expression. Data are expressed as mean ± SEM, * *p* < 0.05.

To clarify the relationship between ageing and non-canonical WNT-5 signaling further, we examined the gene expression of WNT-5A and WNT-5B both at young adult age (average age 24 weeks) and in old mice (average age 50 weeks) (Figure 1A,B). The gene expression levels of the non-canonical ligands WNT-5A and WNT-5B were two-fold higher in PCLS from old WT mice compared to the young adult WT mice, and their expression was correlated with the senescence marker p16 (Figure 1C,D).

### 3.2. WNT-5 Impedes the Expression of Alveolar Epithelial Cell Markers in Whole Lung Tissue

**Figure 2:**
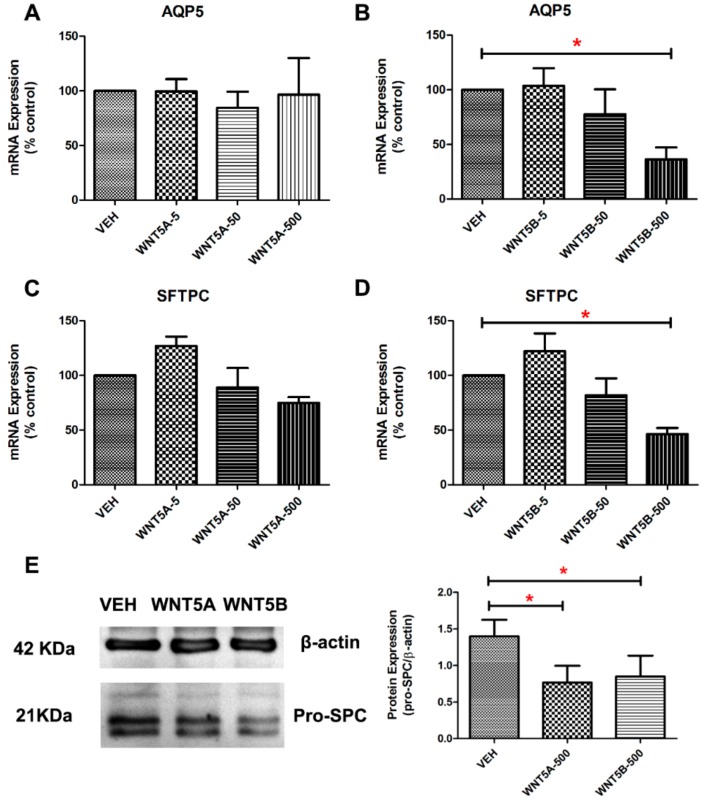
WNT-5A and WNT-5B decreased the expression of alveolar epithelial cell markers in precision-cut-lung slices (PCLS). (**A**–**B**) Normalized mRNA expression level of Aquaporin 5 (AQP5) in PCLS treated with different concentrations of recombinant WNT-5A or WNT-5B (0-, 5-, 50-, 500 ng/mL). (**C**–**D**) Normalized mRNA expression level of surfactant protein C (SFTPC) in PCLS treated with different concentrations of recombinant WNT-5A or WNT-5B (0-, 5-, 50-, 500 ng/mL). (**E**) Representative Western blot of the protein level of pro-surfactant protein C in PCLS treated by recombinant WNT-5A (500 ng/mL) and WNT-5B (500 ng/mL). Bands were quantified by densitometry in 3 separate experiments. Data are expressed as mean ± SEM, * *p* < 0.05.

How these ligands of the non-canonical WNT signaling pathway impact on lung epithelial progenitor cells is still unclear. Thus, we examined the impact of WNT-5A and WNT-5B on alveolar epithelial marker gene expression in PCLS derived from young adult WT mice. The gene expression levels of Aquaporin 5 (Aqp5, marker for alveolar type I cells) and Surfactant protein C (Sftpc, marker for alveolar epithelial type II cells) were decreased after treatment with recombinant WNT-5B (500 ng/mL), but not with WNT-5A (Figure 2A–D). Protein expression of pro-SPC in PCLS was significantly decreased by both WNT-5A and WNT-5B (Figure 2E,F).

### 3.3. WNT-5A and WNT-5B Repress Lung Organoid Formation

**Figure 3:**
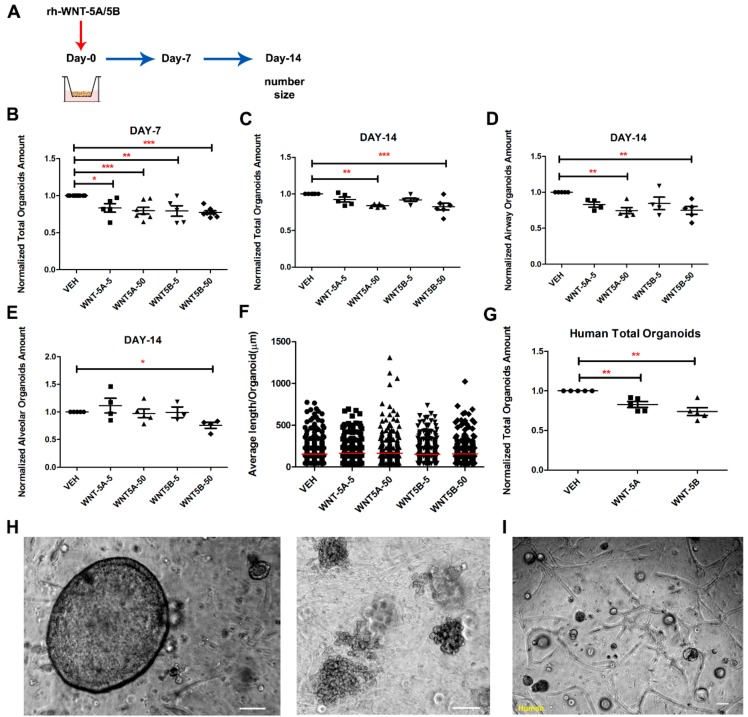
WNT-5A and WNT-5B repressed lung organoids formation and differentiation. (**A**) Schematic of mouse organoid experimental design. (**B**) Quantification of total murine organoids on day 7 treated with recombinant WNT-5A or WNT-5B (0-, 5-, 50 ng/mL), Student *t*-test. (**C**–**E**) Quantification of total, airway type, and alveolar type murine organoids on day 14 treated with recombinant WNT-5A or WNT-5B (0-, 5-, 50 ng/mL), Student *t*-test. (**F**) Quantification of the murine sizes of total organoids measured on day 14 with different treatments of recombinant WNT-5A or WNT-5B (0-, 5-, 50 ng/mL). n > 330 organoids per group from N = 5 independent isolations, one way ANOVA test. (**G**) Quantification of total human organoids treated with recombinant WNT-5A (50 ng/mL) or WNT-5B (50 ng/mL), Student *t*-test. (**H**) Representative light microscopy image of murine organoids at day 14. Scale bar = 100 μm. Airway type of organoids (left), Alveolar type of organoids (right). (**I**) Representative light microscopy image of human organoids at day 14. Scale bar = 100 μm. Data are expressed as mean ± SEM, * *p* < 0.05, ** *p* < 0.01, *** *p* < 0.001.

**Figure 4:**
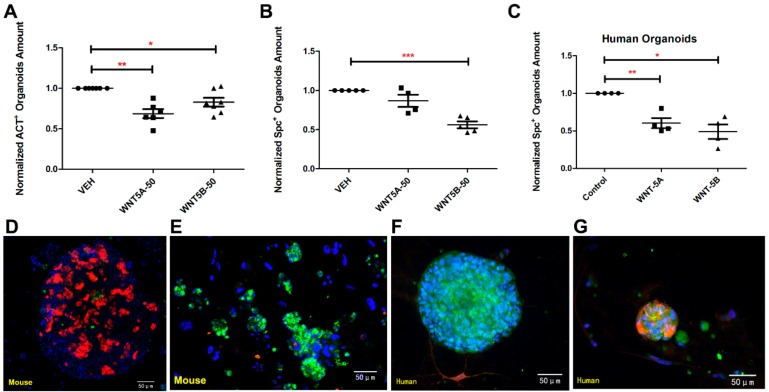
WNT-5A and WNT-5B decreased the amount of acetylated-α tubulin^+^ (ACT^+^) organoids and prosurfactant Protein C (pro-SPC^+^) organoids. (**A**) Effect of WNT-5A and WNT-5B on the murine organoids expressing ACT (**B**) Effect of WNT-5A and WNT-5B on the murine organoids expressing pro-SPC. (**C**) Effect of WNT-5A and WNT-5B on the human organoids expressing pro-SPC. (**D**–**G**) Representative immunofluorescence images of murine organoids on day 14, blue (DAPI), green (pro-SPC), red (ACT). Scale bar = 50 μm. Data are expressed as mean ± SEM, Student *t*-test, * *p* < 0.05.

To understand the specific roles of WNT-5A and WNT-5B on alveolar epithelial cells, we established a lung organoid model derived from a co-culture of epithelial cells (EpCAM^+^/CD45^−^/CD31^−^) and murine lung fibroblasts (CCL206). Previously, we showed that such co-culture yields organoids that consist entirely of epithelial structures, of which ~70% is positive for the type 2 marker pro-SPC and ~10% is positive for the airway marker acetylated-tubulin; the remaining organoids are either double negative or double positive [37]. After ex vivo culture for 7 days and 14 days, the total amount of organoids was significantly reduced by both WNT-5A and WNT-5B (5 and 50 ng/mL, Figure 3B,C), respectively. Compared to the lung slice model, the organoids were more sensitive to WNT-5A and WNT-5B as 50 ng/mL of WNT-5A and WNT-5B already significantly reduced the number of airway type organoids, whereas 50 ng/mL of WNT-5B selectively decreased the number of alveolar type organoids (Figure 3D,E). Neither WNT-5A nor WNT-5B stimulation affected the size of lung organoids measured on day 14 (Figure 3F). To further explore the roles of WNT-5A and WNT-5B on human lung alveolar epithelial progenitor cells, a similar organoid model was set up by co-culturing epithelial cells (EpCAM^+^/CD45^−^/CD31^−^) derived from human tissue. The total amount of organoids was decreased by both WNT-5A and WNT-5B after culturing for 14 days ex vivo (Figure 3G). Immunofluorescence studies confirmed that the number of murine acetylated-α tubulin^+^ (airway type organoids marker) organoids was significantly decreased by both WNT-5A and WNT-5B, however, the number of pro-SPC^+^ (alveolar type organoids marker) organoids was significantly reduced by WNT-5B treatment only (Figure 4A,B). For the experiments using human cells, there were hardly any acetylated-α tubulin^+^ human organoids visible, whereas the number of pro-SPC^+^ human organoids were repressed by both WNT-5A and WNT-5B (Figure 4C). Taken together, these data indicate that both WNT-5A and WNT-5B repress the formation of lung organoids, with WNT-5B having slightly more profound effects on alveolar organoid formation.

### 3.4. WNT-5A/-5B Signaling Repress Canonical WNT/β-Catenin Signaling

**Figure 5:**
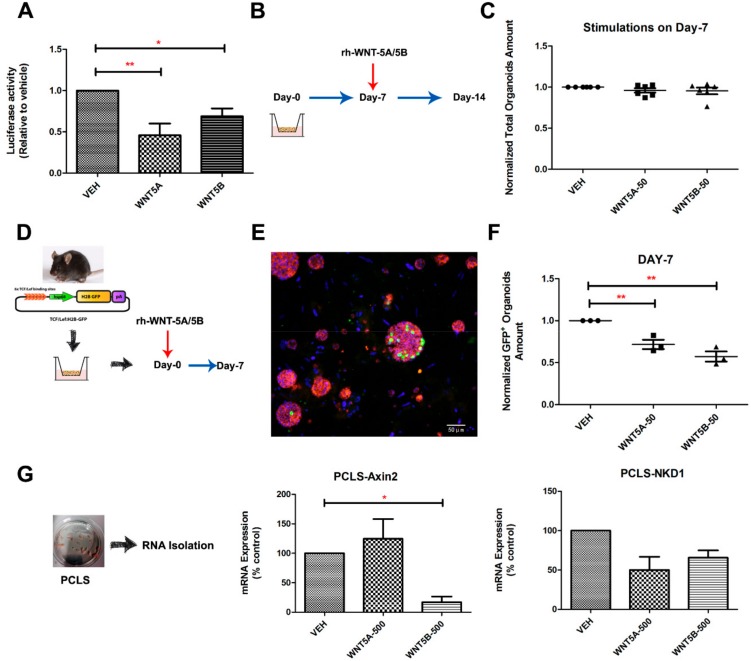
WNT-5A and WNT-5B impaired the WNT/β-catenin signaling in murine lung. (**A**) WNT-5A attenuated the β-catenin-dependent gene transcription (TOP/FOP-flash assay) in human alveolar epithelial cells. N = 4 experiments, one-way ANOVA analysis. (**B**) Schematic of mouse organoid experimental design with a late (start on day 7) stimulation of WNT-5A or WNT-5B (0-, 50 ng/mL). (**C**) Quantification of total murine organoids on day 14 with delayed stimulations of WNT-5A or WNT-5B (0 and 50 ng/mL). (**D**) Schematic of experimental design with TCF/Lef: H2B-GFP transgenic mice. (**E**) Quantification of GFP^+^ organoids on day 7 treated with recombinant WNT-5A or WNT-5B (0 and 50 ng/mL). (**F**) Representative images of GFP^+^ organoids (WNT responsive organoids) from WNT-5A and WNT-5B stimulations, green (TCF:GFP), blue (DAPI). (**G**) Normalized gene expression of AXIN2 and NKD1 on murine lung slices treated with WNT-5A or WNT-5B (0 and 500 ng/mL). Data are expressed as mean ± SEM, * *p* < 0.05, ** *p* < 0.01.

WNT/β-catenin signaling has been recently shown to be essential for adult alveolar epithelial cell progenitors [33,38]. This indicates that inhibition of β-catenin signaling represses, whereas activation of β-catenin signaling promotes alveolar organoid growth. Moreover, we recently demonstrated that WNT-5A negatively regulates WNT/β-catenin signaling and alveolar epithelial cell repair [30], thus here we further explored the mechanism by which WNT-5A and WNT-5B negatively regulate alveolar epithelial progenitor cells, we first investigated their effects on TOP/FOP-flash activity in the alveolar type 2 cell line A549. The activation of β-catenin-dependent gene transcription was attenuated by both WNT-5A and WNT-5B in human alveolar epithelial cells (Figure 5A). We next added WNT-5A and WNT-5B only from day 7 onwards. The number of organoids quantified on day 14 showed no differences between the groups (Figure 5B,C), indicating that the negative role of WNT-5A/5B on alveolar progenitors takes place during the early stages of organoid initiation. To directly identify whether the impact of WNT-5A/5B on alveolar epithelial progenitors is indeed due to down-regulated canonical WNT/β-catenin signaling, we performed an organoid assay using EpCAM^+^/CD45^−^/CD31^−^ cells isolated from TCF/Lef: H2B-GFP mice [39], which harbor a nuclear tagged GFP under the control of the β-catenin sensitive TCF/Lef promoter (Figure 5D). The number of GFP^+^ (WNT responsive) organoids quantified on day 7 was decreased significantly by treatment with WNT-5A and WNT-5B (Figure 5E,F). Consistent with this finding, the gene expression of Axin2 and Nkd1 were decreased in lung slices treated with WNT-5A and WNT-5B (Figure 5G). This support the hypothesis that the canonical WNT/β-catenin signaling is disturbed by WNT-5A/5B signaling, thereby repressing the growth of alveolar epithelial progenitor cells.

**Figure 6:**
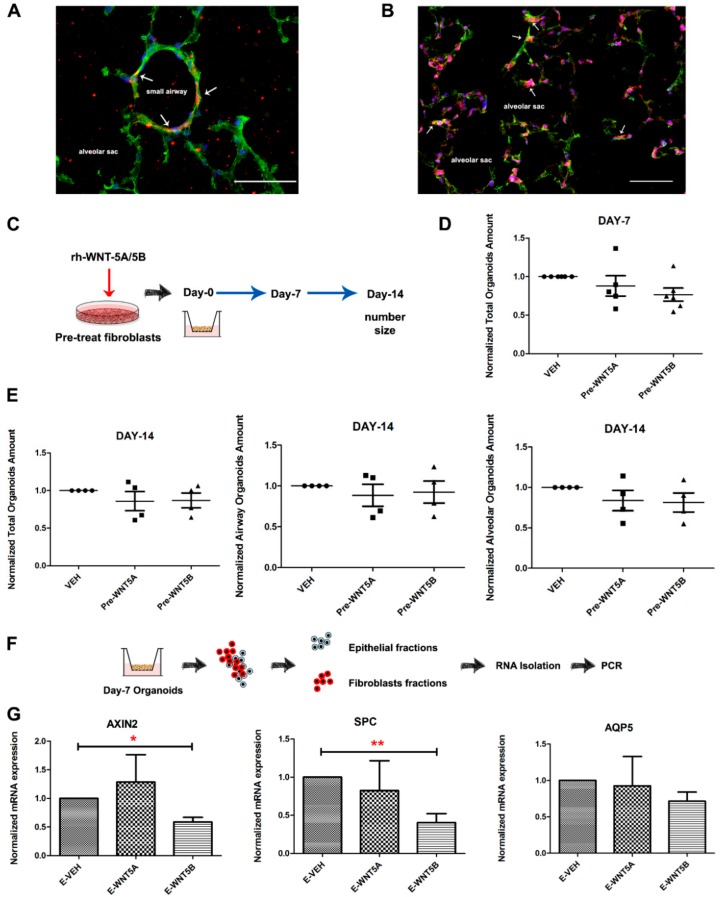
Delayed WNT-5A or WNT-5B signal supported from fibroblasts has no impact on organoids formation. (**A**–**B**) Representative images of murine lung slices stained with WNT-5A (left)/WNT-5B (right) and vimentin. Green (WNT-5A/5B), red (Vimentin), blue (DAPI). (**C**) Schematic of mouse organoid experimental design with the pre-treatment of fibroblasts. (**D**) Quantification of total murine organoids on day 7 generated from co-culture CCL206 fibroblasts pre-treated with WNT-5A or WNT-5B (0 and 50 ng/mL). (**E**) Quantification of total, airway type, and alveolar type of murine organoids on day 14 generated from co-culture with CCL206 fibroblasts pre-treated with WNT-5A or WNT-5B (0 and 50 ng/mL). (**F**) Schematic of experimental design with re-sorting organoids study. (**G**) Normalized gene expression of AXIN2, SPC, and AQP5 on epithelial fractions derived from the organoids cultured 7 days with WNT-5A or WNT-5B (0 and 50 ng/mL). Data are expressed as mean ± SEM, * *p* < 0.05, ** *p* < 0.01.

Immunofluorescence staining on lung tissue showed that WNT-5A or WNT-5B are primarily expressed in fibroblasts in either the small airways or alveolar compartment (Figure 6A,B). This is consistent with data from single cell sequencing of lung organoids [40], showing that WNT-5 is primarily expressed by fibroblasts and less so by the epithelial cells (Figure S1), which express Fzd receptors. In line with this, pre-treatment of the murine fibroblasts with either WNT-5A or WNT-5B for 24 h, followed by extensive washing before introducing them into the organoid assay (Figure 6C) had no effects on organoid growth. Organoid number quantified on days 7 and 14 showed no differences between the groups (Figure 6D,E), suggesting that the fibroblasts may be the source but are not the main target of WNT-5A/5B. Next, lung organoid co-cultures grown in the presence of WNT-5A/5B were enzymatically dissociated, and the fibroblast fractions as well as the EpCAM^+^ cells were individually harvested via MACS-separation. Consistent with the epithelial cell being the target cell, gene expression of Axin2, Spc and Aqp5 was decreased in the EpCAM^+^ fraction aftertreatment with WNT-5B (Figure 6F,G).

## 4. Discussion

Aberrant expression of the non-canonical ligands WNT-5A and WNT-5B has been reported to contribute to inflammation, stem cell ageing, and chronic lung diseases [31,32]; however, their impact on alveolar epithelial repair is unclear. In this study, we demonstrate that WNT-5A and WNT-5B, whose expression is increased in old murine lungs and in COPD [30,31], have divergent repressive effects on alveolar epithelial progenitors. Within the alveolar epithelium where the gas exchange occurs, alveolar epithelial progenitor cells play an essential role in homeostasis, remodeling, and repair of the lung [41,42]. Thus, we established different models including the PCLS, adult mouse, and human lung organoid models to clarify the role of WNT-5A and WNT-5B on alveolar epithelial progenitors. We found that both WNT-5A and WNT-5B repress the alveolar epithelial progenitor cells (AEPs) whereas the differentiation of the AEPs are slightly more influenced by WNT-5B, compared to WNT-5A. Moreover, we found WNT/β-catenin signaling is repressed by both WNT-5A and WNT-5B. Thus, fibroblast-derived WNT-5A/5B repress WNT/β-catenin signaling in alveolar epithelial progenitors, which may serve a physiological role to restrain excessive progenitor cell activation. However, increased expression of WNT-5A/B may contribute to the defective lung repair in COPD and ageing through such a mechanism.

Increasing evidence shows an essential role for WNT/β-catenin signaling in AEPs in supporting alveolar organoid formation [34,43,44]. Indeed, our own findings confirm this and show that inhibitors of WNT/β-catenin signaling repress, whereas activators of WNT/β-catenin signaling (GSK3i) promote, alveolar organoid formation. Since WNT-5A and WNT-5B have previously been reported to repress functional WNT/β-catenin signaling, we thus hypothesized that there would be repressed WNT/β-catenin signaling in response to WNT-5A/B, explaining the growth repression of the organoids. To investigate this, we performed an organoid assay using TC/Lef reporter mice. We found that the number of WNT responsive cells in organoids derived from alveolar epithelial cells was strongly impaired in the presence of WNT-5A or WNT-5B. Reduced canonical WNT/β-catenin signaling was confirmed by the reduced Axin2 expression in alveolar epithelial cells, PCLS, and adult lung organoids. WNT/β-catenin signaling is important for organoid initiation during the first few days of culture [40], but dispensable for organoid growth in the subsequent proliferative phase. Indeed, when added with delay (from day 7 onwards), WNT-5A or WNT-5B failed to affect the growth of epithelial organoids. When added from day 0, WNT-5A and WNT-5B affected organoid number but not organoid size. This indicates that WNT-5A/5B impairs the initial formation of alveolar organoids but not their subsequent proliferation, which is in line with specific repressive effects on WNT/β-catenin signaling, which promotes organoid initiation but not the subsequent proliferative expansion.

In alveoli, fibroblasts are in intimate contact with alveolar epithelial cells and support AT2 maintenance, constituting an alveolar stem cell niche [17]. Since these fibroblasts are characterized by abundant WNT-5A and WNT-5B expression, it was previously suggested that WNT-5A and WNT-5B would support alveolar epithelial repair [33]. Our data support the idea of fibroblasts being a source, and epithelial cells being a target, for WNT-5A and WNT-5B (Figure S1); however, our data do not indicate supportive roles for these WNT ligands in alveolar epithelial organoid formation. Our studies on the epithelial cell fractions dissociated from the organoids cultured in the presence of WNT-5A/5B shows that the expression of AXIN2 and NKD1—target genes for WNT/β-catenin signaling—are decreased. This strengthens the idea that WNT-5A and WNT-5B are repressors but not the activators of the WNT/β-catenin signaling in alveolar epithelial cells. In further support, in the study of Baarsma et al., it was shown that fibroblast-derived WNT-5A attenuates the canonical WNT/β-catenin signaling in alveolar epithelial cells both in vitro and in vivo [30].

Pretreatment of fibroblasts with WNT-5A/5B before co-culture with epithelial cells in organoids was not able to repress alveolar epithelial cells growth, suggesting epithelial cells, and not fibroblasts are the target cell for WNT-5A/B for this effect. We previously demonstrated that ROR2, the WNT co-receptor involved in repression of WNT/β-catenin signaling by WNT-5, is expressed in epithelial cells but not in lung fibroblasts [21,35]. The cell-type specific binding between WNT proteins and receptors such as FZD and ROR may able to activate diverse signaling pathways, which would be of value to investigate in the future. Ahmad Nabhan et al. demonstrated that WNT5A^+^ fibroblasts co-express other WNTs, such as WNT-2, WNT-4, or WNT-9 [33], suggesting multiple WNT activities may be present in the fibroblast population to support cellular interactions with epithelial cells. Besides, Zepp et al. revealed that there are different populations of Axin2^+^ AT2 cells with different levels of WNT responsiveness [45]. Thus, we assume a multiple WNT cascades may functioning simultaneously within the alveolar epithelial niche. Tight control of supportive and repressive WNT signals from lung fibroblasts may serve a physiological function to promote alveolar epithelial progenitors when needed, yet preventing excessive activation of these cells in homeostasis. It is of great interest to further explore the WNT dynamics contributing to lung repair to understand how the imbalance of canonical and non-canonical WNT signaling pathways contribute to defective lung repair in the ageing lung and in COPD.

WNT-5B, which is related to WNT-5A containing 80% similarity in mammals, is poorly studied unlike WNT-5A. Van Dijk et al. identified that the WNT-5B activated non-canonical WNT signaling pathway is involved in inducing tumor invasion [32]. Besides, the expression level of WNT-5B in airway epithelial cells from COPD patients was reportedly increased by cigarette smoke, activating TGF-β/smad3 signaling downstream of WNT-5B [31]. We focused our study on the alveolar epithelium, and demonstrate that WNT-5B inhibited the growth of the alveolar epithelial progenitors but also repressed their differentiation. It would be of interest to study how such a mechanism contributes to chronic lung diseases in the future. Evidence has accumulated that both WNT-5A and WNT-5B are involved in the development and progression of chronic lung diseases, including asthma, IPF, lung cancer, and COPD [13,15,30,35,40,46]. A mechanistic understanding of how defective WNT signaling underpins these diseases is necessary to pave the way to clinical applications such as drugs or cell therapies that correct the WNT signaling imbalance. Our findings indicate that WNT5A/5B mediated non-canonical WNT signaling inhibits lung epithelial progenitors. As their expression is increased in ageing and in COPD, they might be potential drug targets.

## 5. Conclusions

Collectively, WNT-5A and WNT-5B repress the growth of lung organoid co-cultures of fibroblasts and epithelial progenitors, with WNT-5B preferentially restraining both the growth and differentiation of the alveolar epithelial progenitors. We provide evidence that WNT-5A/5B results in the inhibition of WNT/β-catenin signaling in alveolar epitheliums. Targeting the imbalance of the canonical and non-canonical WNT signaling pathway in the lung may help restore alveolar repair in the ageing lung and in COPD.

## Supplementary Materials

The following are available online at https://www.mdpi.com/2073-4409/8/10/1147/s1, Figure S1: Expression of WNT-5A and WNT-5B on fibroblasts and EpCAM^+^ cells. Table S1: Primers used for RT-PCR. Table S2: Antibodies used in this study.
