# Supplementary material for: Mesenchymal WNT-5A/5B Signaling Represses Lung Alveolar Epithelial Progenitors
Source: Cells. 2019 Sep 25;8(10):1147. doi: 10.3390/cells8101147 (PMC6829372; doi:10.3390/cells8101147)
Supplement: Supplementary file 1 [file cells-08-01147-s001.pdf]

## Supplementary materials

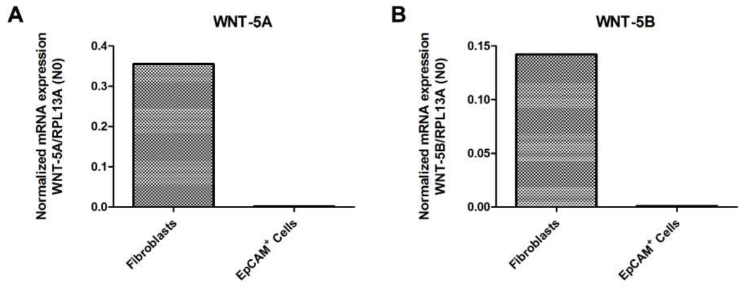


**Figure S1**. Expression of WNT-5A and WNT-5B on fibroblasts and EpCAM$^+$ cells. A. mRNA expression of WNT-5A on CCL-206 mouse fibroblasts and EpCAM$^+$ cells. B. mRNA expression of WNT-5B on CCL-206 mouse fibroblasts and EpCAM$^+$ cells.

**Table S1. Primers used for RT-PCR**

| Name | Species | | Sequence |
|---|---|---|---|
| 18S | Mouse | Forward | AAACGGCTACCACATCCAAG |
| | | Reverse | CCTCCAATGGATCCTCGTTA |
| AXIN2 | Mouse | Forward | CAGTGAGCTGGTTGTCACCT |
| | | Reverse | TCCTCAAAAACTGCTCCGCA |
| AQP5 | Mouse | Forward | CTTGTGGGGATCTACTTCACCG |
| | | Reverse | AAGTAGAGGATTGCAGCCAGG |
| B2M | Mouse | Forward | ACCGTCTACTGGGATCGAGA |
| | | Reverse | TGCTATTTCTTTCTGCGTGCAT |
| NKD1 | Mouse | Forward | TAGACCTGGCGGGGATAGAG |
| | | Reverse | GTCAAGGAGGTGGAAGGAGC |
| RPL13A | Mouse | Forward | AGAAGCAGATCTTGAGGTTACGG |
| | | Reverse | GTTCACACCAGGAGTCCGTT |
| SFTPC | Mouse | Forward | GGAGCACCGGAAACTCAGAA |
| | | Reverse | GGAGCCGCTGGTAGTCATAC |
| WNT-5A | Mouse | Forward | CTGCGGAGACAACATCGACT |
| | | Reverse | TACAGGAGCCAGACACTCCA |
| WNT-5B | Mouse | Forward | AGTTTGTGGATGCCCGAGAG |
| | | Reverse | CAGGCGACATCAGCCATCTT |

**Table S2. Antibodies used in this study**

| Primary antibodies | | | |
|---|---|---|---|
| **Name** | **Host** | **Company** | **Dilution** |
| β-actin | Mouse | Sigma (A5441) | 1:500 |
| Pro-SPC | Rabbit | Millipore (AB3786) | 1:200 |
| Acetylated α tubulin | Mouse | Sigma (T7451) | 1:200 |
| WNT-5A | Mouse | R&D Systems (AF645) | 1:50 |
| WNT-5B | Goat | Santa Cruz (sc-109464) | 1:50 |
| Vimentin | Mouse | Dako (M0725) | 1:100 |
| Secondary antibodies | | | |

| Name | Host | Company | Dilution |
|---|---|---|---|
| Anti-rabbit Alexa Fluor 488 | Donkey | Thermo Fisher (A21206) | 1:200 |
| Anti-mouse Alexa Fluor 568 | Donkey | Thermo Fisher (A10037) | 1:200 |
| Anti-mouse Alexa Fluor 488 | Donkey | Thermos Fisher (A21202) | 1:2000 |
| Anti-goat Alexa Fluor 568 | Donkey | Thermos Fisher (A11057) | 1:2000 |
